# Funders: The missing link in equitable global health research?

**DOI:** 10.1371/journal.pgph.0000583

**Published:** 2022-06-03

**Authors:** Esmita Charani, Seye Abimbola, Madhukar Pai, Olusoji Adeyi, Marc Mendelson, Ramanan Laxminarayan, Muneera A. Rasheed

**Affiliations:** 1 Division of Infectious Diseases & HIV Medicine, Department of Medicine, Groote Schuur Hospital, University of Cape Town, Cape Town, South Africa; 2 National Institute for Health Research Health Protection Research Unit in Healthcare Associated Infections and Antimicrobial Resistance, Imperial College London, London, United Kingdom; 3 Department of Infection Control and Epidemiology, Amrita Institute of Medical Science, Amrita Vishwa Vidyapeetham, Kochi, Kerala, India; 4 School of Public Health, University of Sydney, Sydney, New South Wales, Australia; 5 Julius Global Health, Julius Center for Health Sciences and Primary Care, University Medical Center, Utrecht University, Utrecht, The Netherlands; 6 School of Population and Global Health, McGill University, Montreal, Québec, Canada; 7 Resilient Health Systems, Washington, DC, United States of America; 8 Johns Hopkins Bloomberg School of Public Health, Baltimore, MA, United States of America; 9 Center for Disease Dynamics, Economics & Policy, New Delhi, India; 10 High Meadows Environmental Institute, Princeton University, Princeton, NJ, United States of America; 11 Independent Consultant, Karachi, Pakistan; University of Sao Paulo: Universidade de Sao Paulo, BRAZIL

## Abstract

Global health research is mired by inequities, some of which are linked to current approaches to research funding. The role of funders and donors in achieving greater equity in global health research needs to be clearly defined. Imbalances of power and resources between high income countries (HICs) and low- and middle-income countries (LMICs) is such that many funding approaches do not centre the role of LMIC researchers in shaping global health research priorities and agenda. Relative to need, there is also disparity in financial investment by LMIC governments in health research. These imbalances put at a disadvantage LMIC health professionals and researchers who are at forefront of global health practice. Whilst many LMICs do not have the means (due to geopolitical, historical, and economic reasons) for direct investment, if those with means were to invest more of their own funds in health research, it may help LMICs become more self-sufficient and shift some of the power imbalances. Funders and donors in HICs should address inequities in their approach to research funding and proactively identify mechanisms that assure greater equity–including via direct funding to LMIC researchers and direct funding to build local LMIC-based, led, and run knowledge infrastructures. To collectively shape a new approach to global health research funding, it is essential that funders and donors are part of the conversation. This article provides a way to bring funders and donors into the conversation on equity in global health research.

## Introduction

Global health in its broadest term is defined as "the area of study, research and practice that places a priority on improving health and achieving equity in health for all people worldwide” [1]. In this article we focus on the current disparities in global health research, focusing on inequities and power asymmetries in current research structures and funding mechanisms. These inequities in research are reflected in inequities in global health outcomes. Researchers and journals within the global health community have been increasingly vocal regarding these inequities contributing to the discourse about what is wrong with global health [2, 3]. The epistemic injustices in global health research funding are shaped by deeply entrenched unequal power dynamics, rooted in a colonial history that has to date consistently applied an outsider gaze to local problems and needs [4]. For their part, funders and donors, though they have diverse platforms for expressing their voice, often in ways that go unchallenged, are not engaged in this discourse, nor is there any evidence of their reflection on their responsibility, accountability and positionality in global health research and the injustices that perpetuate in this space. This, together with the lack of transparency in the decision-making process in funding is a critical blind spot [5, 6].

There have been calls for example for urgent review of the current funding mechanisms in the UK research environment specifically in global health research collaborations with low- and middle-income countries (LMICs) [7]. The need to align the positionality and gaze of global health funding models has been described, providing recommendations for how to address the power imbalances which inhibit epistemic diversity [4]. This approach calls for greater division of power between North and South actors which will require active engagement on both sides. It is important to note that inequities in research and in health outcomes are linked to deeper and sustained inequities in the structural determinants of the global political economy that affect both health and research. Within high income countries (HICs) too, the racial and socioeconomic status of populations impacts their access to health and health outcomes, with research into their health needs often under-resourced [8].

Understanding and changing the status quo requires closer examinations of the strategic goals and incentives that drive funders’ decisions, and influences what they fund. It also requires engaging with funders in HICs, and potential funders in LMICs in conversations that aim to collectively shape a new approach to global health research funding. Adding to the existing discourse on this topic, we describe the current drivers of injustice and the complicity of different actors working in systems designed to perpetuate a division of labour that is highly detrimental to those who hold less power, the majority of whom are in LMICs.

## Current challenges in funding global health research

The current power asymmetries influence control of resources and knowledge generation and prioritisation [4]. Global health research funding primarily comes from HICs and may not correlate with the burden of disease [9]. Major donors such as the US and European country governments, and philanthropies like Bill & Melinda Gates Foundation (BMGF), the World Bank and Global Fund are highly influential in shaping the global health agenda [10]. When HIC donors give grants, they predominantly fund institutions, contractors, and principal investigators in their own countries. For example, 70% of Fogarty grants go to US and HICs, 73% of Wellcome Trust grants support UK-based activity, 80% of USAID contracts go to US firms, and 88% of grants by the BMGF is estimated to be held by global North institutions [11–14].

In the development and humanitarian sector, a large proportion of funds are granted to global North non-governmental organisations (NGOs) [15]. This concentration of funds in HICs gives them power to dictate what research is done, how it is done, who carries out the research, ownership of the data and knowledge generated, and subsequent representation on research outputs. Hence, the authorship on global health publications is heavily skewed towards HIC researchers who are often first or senior authors [16]. Parachute research is also a likely consequence of the power dynamics inherent in research funding. This domination is also seen in global health leadership. Reports by Global Health 50/50 indicate the lack of diversity in global health leadership with potential impact on priorities and policies. In the latest Global Health 50/50 report, more than 70% of global health leaders were men and half came from just two countries: UK and US. Moreover, only 5% of women from LMICs were in leadership roles [17, 18].

The topic of global health research differentially benefits populations of the global North [4, 19]. Examples of such inequities include the trickle-down science of adopting technology from HICs to LMICs, often with limited consideration for the contextual needs of LMICs [20]. While there are common interests (between funders and grantees) in generating and sharing new knowledge, there are divergent interests in the distribution of power. The asymmetry of power and voice is a design feature of the system, not a bug. Disrupting the asymmetries in these systems will need active reflexivity and participation from funders, who to date due to their privileges have been shielded from the effect of injustices which shape the global health environment. The critical question is how to make such disruption to the system palatable and acceptable, as to those who hold power within the system it is working as it should. The first step is to create an environment where there is ownership, participation, and equity [4, 21, 22].

The power asymmetries translate to institutions and investigators in HICs being the major recipients of funds to implement research addressing health inequities in LMICs [13]. For example, malaria and TB primarily impact LMICs, but research funding is mostly held by HIC institutions [14, 23]. In the case of TB, whilst LMICs account for 98% of reported cases, the largest funder remains the USA National Institute for Health which by 2020 had awarded 93% of its allocated TB research budget of $339 million to US-based institutions [24]. The BRICS (Brazil, Russia, India, China, and South Africa) countries jointly account for 47% of worldwide TB cases. Whilst it is not possible to account for their total contributions, investment in TB research from all these countries declined in 2020 [24].

The argument is often made that LMIC researchers do not have the capacity to deliver global health objectives unaided. This is an inappropriate generalisation. There are many laboratories in LMICs for example, capable of high-quality research, and other LMIC laboratories could come up to the same standard, given time and appropriate investment. Furthermore, global health research is not just about technical skills in science, it is also about scaling evidence, fighting systems that perpetuate inequities and injustice, social trust, effective leadership, and the administrative challenges of managing large grants. These skills require scientific credibility, but also local and contextual knowledge and experience [25]. HIC scientists who persist with a ‘saviour’ mentality, while doing very well from being affiliated with LMICs for their own career advancement, often remain unchallenged in presenting LMIC researchers as insufficiently skilled, while ignoring home-grown LMIC opportunities for innovation [26]. This is driven by imbalances in economic and cultural power between HIC and LMIC scientists, which confer unchecked privileges to those in HICs [27]. HIC scientists are better trained in navigating the academic research environment (e.g. grant writing and publishing), which is historically anchored in the global North. This translates into the distinct underrepresentation of LMIC researchers on grant applications. The underrepresentation of LMIC partners is also a continued problem with funding panels [25]. Equally, the persisting mentality of victimhood by many LMIC professionals needs challenging, as does the abdication of responsibility and underinvestment by leaders of many LMICs, a practice that perpetuates dependency of the South on the North [28, 29].

Historically movements for change have been led by those affected–the disenfranchised leading the US Civil Rights Movement, women revolting against patriarchy, families with children with disabilities setting up organisations to serve children with similar issues. Those movements presented credible threats that beneficiaries of the status quo could not ignore. Global health research may need the same. To redress the power imbalances, LMICs need to participate not as supplicants, but as increasingly assertive partners who co-finance a joint enterprise. This expectation is however, complicated by geopolitical and economic drivers [30]. First, the growth of philanthro-capitalism–a way of doing philanthropy which mirrors for-profit business–driven by global rule systems, tax rules, transnational corporate influences, the growth of commercial interests in the health sector, and the focus on a biotech paradigm which impact HIC motivations for occupying space in global health [31]. For example, the BMGF emphasis on technology may be driven by market forces in HICs and overlook the socio-cultural determinants of health [32]. Second, the role of debt servicing in shrinking the fiscal space for health in many LMICs cannot be ignored. In 2019, over 30 African countries spent more on debt servicing than on health [33]. Most LMICs cannot afford to finance health research within their own countries. Third, universal expectations on politically and economically disparate countries that comprise LMICs is not just or appropriate and in many ways reinforces the narrative of the powerful. This too is occurring at a critical time when decisions in what to prioritise and fund in global health are made without equitable representation of LMICs actors [34].

On the other hand, the role of private actors in global health is increasing. For example the Global Fund involves a multiplicity of private actors in a variety of ways in co-responsibility for and co-development of policies and interventions [35]. This increasing power is not necessarily aligned with how much private actors are investing in global health, which in the case of the Global Fund amounts to 5% of the total investment, bringing into question the disproportionate power that private actors have in global health despite low investment [35]. It also raises the question of the interest of private actors in global health, given that the corporate and commercial determinants of health tend to affect LMICs disproportionately [36, 37].

With variations across countries, factors affecting the capacities of LMICs to self-finance greater portions of their health expenditures include the sizes of their economies; the tax-to-GDP ratio, which is very low in some countries (and important because governments can spend only the revenues they collect in taxes, unless they borrow from local or foreign sources); the choices they make within their budget constraints (reflecting their priorities); and revenue lost to corruption. LMICs that can afford to, do finance their own research. The BRICS countries are one such example. With growing local investment in its scientists and institutions, China has taken over from US as the leading country in terms of scientific output [38]. China, South Africa, and India are increasingly assertive and can provide examples for others in fighting for and earning greater clout. We need more such examples from the global South. The caveat is that these countries are vast with a young population increasingly gaining higher education. In regions with smaller countries, regional pooling is a viable option. Another way to create a critical mass of researchers is by increasing funding to the global South by investment in and building of more health research institutes. This is essential for developing and sustaining track records in health research and greater recognition of leadership in LMIC-based institutions.

Hence, despite progress in some LMICs, inequities in opportunities and funding generated in LMICs remain a critical issue putting their researchers and institutions at the mercy of HIC funders, contractors, and principal investigators (PIs). One of the reasons for the difficulty in shifting this inequity is the level at which the decision-making process is open to researchers and other collaborators from LMICs. Current systems continue to regard LMIC collaborators as passive recipients or data collectors, with little representation and voice at the decision-making level. By the time LMIC partners are brought into decision making, the priority areas for research are already determined, funds divided, research questions developed and strategy set. This can be explained by the political, economic, and cultural power invested in global health funding remaining concentrated in the global North [17]. Indeed, the current system is working as it was intended to.

Changing current systems requires equity in representation beginning at the sources of financing and continuing at the top table when priorities for global health are identified and funding streams assigned. To achieve meaningful change, both sides of the coin need to be tackled. Firstly, LMICs need to be held to account to invest more of their own funds in health and research, to free their researchers from dependency on donors dominated by HICs. Secondly, HICs donors need to address the existing processes of funding mechanisms that create these inequities e.g., excessive reliance on the leadership of Northern institutions, a practice that reduces Southern researchers to bit players in their own countries.

Given that currently, donors and funders in HICs contribute most to funding global health research, we focus on inequities that arise from this imbalance. The specific questions for donors in LMICs need to be part of a separate piece considering different challenges in very diverse politico-economic contexts. It is important however, to acknowledge that the questions and challenges for donors and funders in HICs are equally applicable to funders in LMICs as power manifests itself in similar ways.

Funders of global health research should consider, engage with, and reflect on, their role in (in)equitable global health research. Firstly, current funding structures were set up for systems where centuries of political stability have created infrastructure for governance, knowledge acquisition, and dissemination (especially for mainstream populations)–such as in the US, Canada, UK, Australia, Sweden, Netherlands and Singapore [34]. Secondly, current systems for identifying funding priorities do not match the challenge of bridging capacity divides between HICs and LMICs–such that within partnerships, LMIC collaborators often remain no more than glorified data collectors [39]. Thirdly, existing governance frameworks and knowledge acquisition and dissemination were developed based on the experiences and interests of mainstream HIC populations. However, motivation for engagement, activism, and effective participation in global health research varies within and across countries depending on individuals’ experiences, perspectives, and privileges. Fourthly, research calls in global health are often biased towards issues that predominantly affect HICs, and when LMICs are targeted, since the panels that develop these calls are often convened by HIC-minded personnel, key LMIC considerations are often lost. Restrictive criteria for participation in the consultations to develop research areas for funding often leave LMIC partners out. Also, research areas emphasized by major philanthropies, such as BMGF, are often focused on technological solutions, rather than health systems strengthening and primary health care [40].

However, there are examples of global health research funding which aims to address inequities. New mechanisms such as the Joint Programming Initiative for Antimicrobial Resistance (JPIAMR) new mechanisms like JPIAMR, allow for a joint review process across many national funders and allow countries to support other country researchers in a multilateral mechanism [41]. The Consultative Group for International Agricultural Research (CGIAR) that operates globally but mostly in LMICs, has been transformational in the agricultural space by creating a mechanism that is responsive to needs globally. Such mechanisms or platforms provide potential ways forward for global health research funding.

## Where are the opportunities for addressing these inequities?

**Fig 1:**
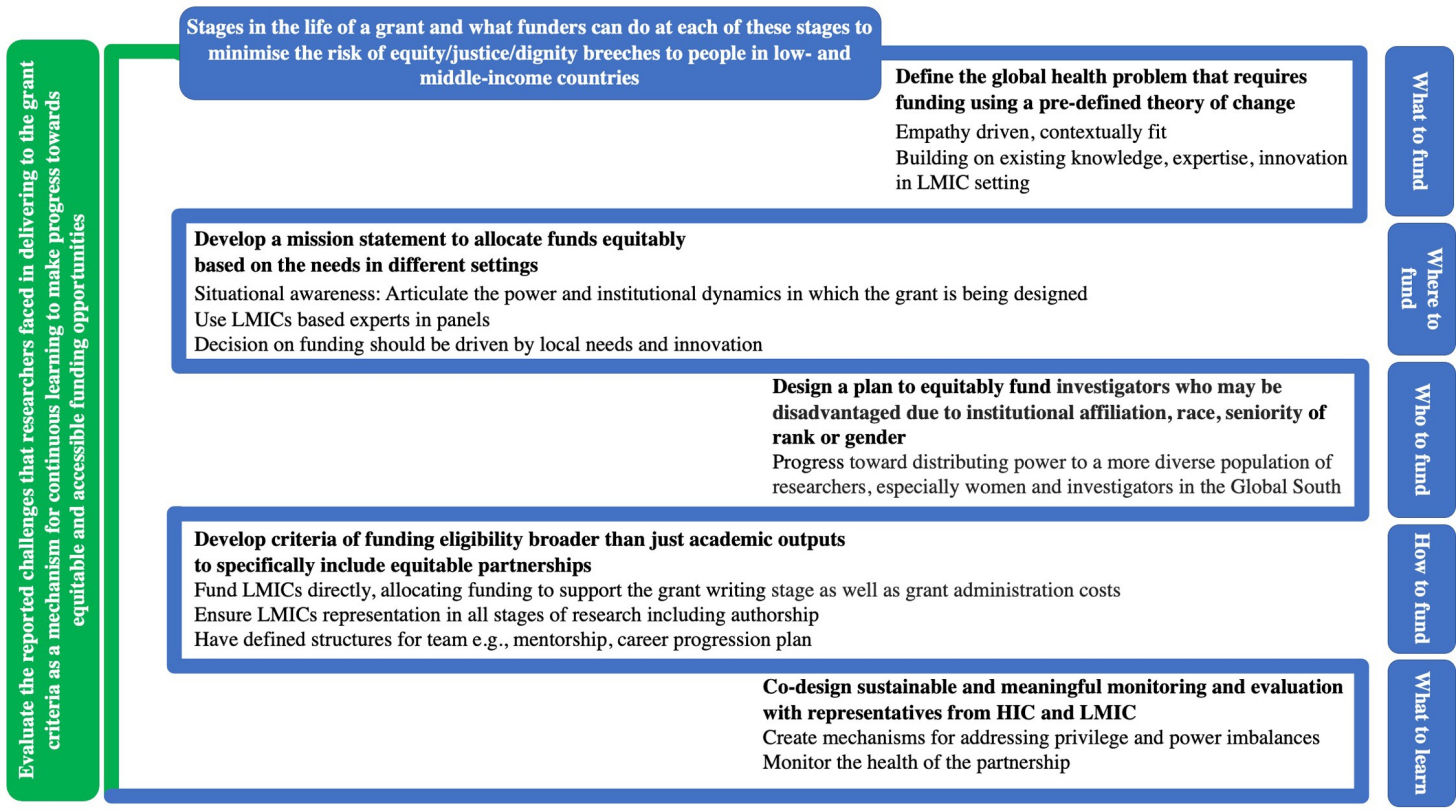
Opportunities for funders to redress the inequities in global health research during the different stages of a grant.

Along the lifetime of a grant, there are missed opportunities for assuring equitable and ethical distribution of funds and resources that speak to the true principles of global health (Fig 1). Considerations for funders to include throughout the life course of grants in global health include:

- Situational awareness: funders need to articulate the power and institutional dynamics in which the grant is being designed, and they need to track who benefits from their funding.
- Develop a mission statement: to support the awardees to ensure equity in research across diverse domains for the life course of the research.
- Equitable allocation of funds: adequate and equitable allocation for all resources including expenses that reflect the needs of different settings, and attention to increased costs for LMICs researchers who may spend periods in HICs.
- Funding structures to uplift the disadvantaged: facilitate an environment where those afflicted with inequities are given sufficient power and resources (through direct funding instead of donor models of grants) to develop their own leadership opportunities to build local capacity and own the research and data generated.
- Capacity-strengthening: opportunities should include reciprocal observerships as well as building bridges between funders and researchers to facilitate LMIC and HIC researchers spending time working with the funder.
- Fostering diversity and inclusion of research teams through the grant cycle: co-development of research; equity in representation of people in outputs including scrutinising authorship, fellowships, conference presentations, and softer outputs such as panels convened for international meetings [6].
- Process of knowledge generation: clarity on ownership of data, interpretive tools (such as methods and frameworks) and the process through which knowledge is generated.
- Reflection and feedback: a nominated HIC and LMIC researcher to work directly with funders to build bridges for communication and learning; and transparency in the reflection and feedback from researchers to learn from mistakes and challenges within existing funding streams.

## Time for donors and funders to reflect

Whilst there have been calls for an ethical framework for reforming the funding processes in global health, to our knowledge there has not been a statement from many funders and donors explicitly engaging with these calls [42, 43]. Meaningful change requires candid conversations across institutional boundaries. What factors make it possible to have initiatives and programmes that primarily benefit research institutions in the global South, such as the Africa Centres of Excellence Project [44] or the Africa Centres for Disease Control and Prevention? We cannot move toward change without listening to the stories of the global South. What are the institutional and individual factors at play? Do they enable or hinder employees seeking to innovate? Or does it take extraordinary effort by employees to experiment outside the status quo? To transform this field what is undoubtedly needed is to have an opportunity for candid conversations with donors and funders as part of the process to transform the field [3].

Specific questions that need reflection and response from donors and funders are: 1) How diverse and inclusive are donors and funders, especially at the leadership level? 2) What do they understand as the goals of global health and how do those goals fit with their institutional priorities? 2) What geopolitical and technocratic factors do they see as drivers of global health? 3) What synergies and conflicts do they see between the first two considerations? 4) What percentage of funding will be directly awarded to global South recipients? 5) What do they see wrong with global health, and why? 6) What are the challenges in acting on the recent recommendations to address power asymmetries and decolonise global health? 7) What support do they envisage they need to bring about progress in this field? Reflecting on these questions will help funders to identify their role in addressing the prevailing injustices in global health.

Funders and donors need to work closely with the global health community across LMICs and HICs to define a pathway in global health research and development to address contextual injustices. This is critical to ensure and accounting for and understanding of the diversity of knowledge systems and paradigms. Through review of the literature and current discourse and discussions with stakeholders we propose a framework of key considerations to be used by funders from inception of funding schemes through to their delivery and monitoring and evaluation (Table 1). This framework can be applied to assess the ownership, partnership, and equity of the funding process providing a reflexive tool for funders to assess themselves. These questions are formulated to help funders reflect on the current injustices that existing systems are perpetuating, with potential solutions which funders can actively apply to disrupt asymmetries and progress towards ownership, participation, and equity.

**Table 1:** Key considerations and metrics for funders towards more equitable global health.

QUESTIONS	KEY CONSIDERATIONS TO REFLEXIVELY CRITIQUE AND MEASURE THEIR OWN PROGRESS	RECOMMENDATIONS FOR FUNDERS
**What should be funded?**	Is there a way to collectively create a theory of change (ToC) for the outcomes that a funding call seeks to achieve? Can the projects funded as part of the process be identified on the ToC?^2^	Transparency in sharing a ToC for potential applicants to be able to navigate the grant specifications and help the funding panel to measure the suitability and selection criteria for applications.
	Who are funders listening to and do they include voices of those with lived experience and expertise from regions where funding is directed to, e.g., low-, and middle-income countries (LMICs)?	Funding bodies’ executive committees, panels, and reviewers must have equitable and diverse representation from LMICs, and individuals from different gender, race, and expertise. For funding calls directed at LMICs, the committees deciding on research priorities and the selection panels should have at least 50% LMIC representation.
	How can we balance inequities in innovation? Simple, organic solutions can be found that are hugely impactful–for example the Friendship Benches to address mental health issues that were first implemented in Zimbabwe [45]. Can we avoid airlifting innovations from one context to another without ensuring they are appropriate to where they are to be implemented or scaled up?	LMIC representation is research prioritisation stage, as above. The evidence gathering for research prioritisation should not be conducted by costly consultancies, but by consultation with LMIC expertise and through evidence-based data. Negative examples include paying hefty sums to consultancies to conduct rapid evidence synthesis, which is often cursory and superficial.
	How do we ensure innovations stem from solidarity and empathy and not sympathy or saviourism?	The need for innovation, the type of innovation, and the point of intervention for innovation needs to be driven by LMIC needs and expertise. All too often it is driven by parachute research, opportunistic researchers in high-income countries (HIC) keen to test their innovations in LMICs. The ultimate aim should be sustainability to end dependency on HIC.
	How can we work towards defining research strategies, proposals and protocols that demonstrate an understanding of context and resource availability and data access incorporating knowledge and innovation already in practice in LMICs, which often deliver same or higher quality care (comparable to high-income countries—HICs) in much more resource-limited settings?	Research proposals need to be scrutinised for use of local data and innovation to inform the research question and design, with specific attention to feasibility in the context they are being implemented.
**Who should do the funding?**	Where is the source of the funding, whether public, private, or a hybrid form of the two and what are the drivers for the funders to engage with the specific funding call?	Practice transparency in declaring source of financing for research funding and identify potential conflicts of interest of the investors. Develop strategies for separating the investors from the funding process to ensure impartial allocation of funds and research prioritisation.
	What level of effort should LMICs make to reduce the power imbalance?	The funding bodies should proactively engage with LMIC actors including funders earlier in the process to have a voice in prioritisation and allocation of funding.
	How would more LMIC-funded research in global health change the North-South power dynamics?	Establish a transparent process for tracking the progress of funding to evaluate the different models on outcomes, e.g., where funding originates from and who the recipients are and the impact of this on outcomes. Exit interviews with all actors is one way to achieve this.
	Which global North entities (public, private) offer good practices worth emulating by others? How can we have an agreement on these practices?	Investigate the current practices and establish a code of ethics for funders in global health to abide by.
**Who should be funded?**	How can we create equitable opportunities for LMIC researchers to be principal investigators, e.g., to receive direct funding (without HIC actors or intermediaries)?	Funders to set up more accessible platforms for LMIC principal investigators to be able to participate in funding calls. This may include establishing more direct links with LMIC institutions and societies.
	How can access to funding streams for investigators based in LMICs be improved?	Greater LMIC representation in funding panels and Committees will bring the topics of concern and the barriers to participation for LMIC colleagues to the attention of funders. This, together with improving access to resources for training including LMIC friendly fees to attend courses will increase access. Making funding calls accessible also includes understanding the need to compensate for tuition fees, visa and travel barriers, and provision of training programmes that meet needs of LMIC based researchers which may be different to HIC researchers. Providing funding for HIC institutions to host visiting scholarships.
	How can we support supervisorship and leadership of research predominantly in LMICs erasing bottlenecks in research and mobilising existing knowledge and innovation?	As above. In addition, supporting through funding partnerships between HIC and LMIC institutions which encourages and enables bidirectional scholarships and research visits. Include contribution to institutional capacity (No. of investigators trained over time) as one of the asks.
	How can funders move away from relying on the reputation of select, established investigators and take more risks to fund new investigators in LMICs?	Recognition of the team efforts in grant applications. Stipulating the role of each co-applicant in funding applications in the same way that some journals stipulate authorship contributions. Opening more funding opportunities for new investigators and investing in supporting successful applicants by partnering through mentorship with established teams or institutions.
	What can be other evaluation criteria, which can complement lack of experience in securing big grants or lack of academic publications?	Acknowledging the diversity in knowledge and paradigms, including: Capacity for effective leadership Generation of new research ideas Partnership with established centres e.g., matching new investigators with established centres The diversity of roles of investigators and their impact on their communities through outreach work, engagement and capacity strengthening, social enterprise, and engagement.
**How should they be funded?**	Where is the money going to? Does the majority of HIC funds remain in HICs?	Have a clear agreement and vision in the ToC on where the funding will be spent. Commit to directly allocating most of global health funding for LMIC research to LMICs. Where funding is to be given to HICs it should be in clear partnerships with LMICs and equitable processes for division of work and outputs agreed to from the beginning of the funding process. Demand to see as part of the evaluation a statement on equitable work ethics and equitable data ownership and publication plans which can be tracked for the duration of the grant.
	Will the funders resource the time and institutional costs of LMIC individuals who are called upon to participate in the process?	Allocate funds for the conceptualisation, writing and submission (and administration) of large grants which will requires substantial effort and time. This is important as not all LMIC institutions recognise the same indicators for academic excellence and many researchers will be working in their own time to write grants, having access to fewer resources and with less experience than HIC partners. For large international collaborations, provide seed funding for bilateral visits to develop research ideas.
	Can ensuring a mix of academic and practitioner experience be a way to balance principal investigators on collaborative and consortia grants? Can we ensure due recognition of leadership of investigators in LMICs e.g., to not facilitate parachute models of research?	Funders should stipulate for evidence of equitable representation and inclusion of LMIC investigators in leadership positions in grants with a transparency declaration on roles in the grant process. Ensure diversity of the LMIC investigators e.g., with respect to age, gender, and experience.
	How can greater representation of LMIC authors on research outputs especially as first and last authors be incentivized? Can being on publications in which they are neither first or last author it be a key consideration for the HIC investigators when securing grants or be on the review panel of grant applications?	Establish standardised rules of conduct for representation. For example, for HIC-LMIC partnerships the authorship and data ownership agreements should be in the application, with one solution that HIC-LMIC authors are equally represented as first and last authors, and this then will define the division of work and labour. Additionally, it will enable a space for new investigators to grow and learn. Data collected in LMICs should generally be owned by LMIC institutions and these institutions should be encouraged to take the lead on writing opportunities for their research staff.
	What can be some metrics that look at value for capacity strengthening beyond high impact publications and which are also valued by academic institutions?	Individual and institutional capacity strengthening criteria should be agreed to and be part of the funding call. The various means of capacity strengthening, and its measurement include: The career progression of LMIC grant participants e.g., fellowships, studentships, promotions through predominantly in-house career plans and mentorship Opportunities for bi-directional learning and travel as visiting researchers to north-south and south-south institutes Presentation in international conferences and meetings Position in authorship Training provided
**What to learn?**	What mechanisms can be put in place to proactively deal with issues arising due to power asymmetries?	Stipulate a code of conduct and leadership that is equitable for the lifetime of the grant. Establish safe mechanisms for co-applicants to be able to raise concerns regarding abuse of power. This must be anonymous, and impartial and ideally managed by the funder. Conduct exit interviews with awardees to identify any problems for learning for future grant calls. Currently there is no mechanism for providing feedback to funders regarding problems. This needs investment from funders.
	Is there reciprocity in leadership, availability of opportunity for training, access to sites, and knowledge being generated?	The funding criteria must include: Opportunities for bi-directional learning and travel as visiting researchers to north-south and south-south institutes; including funding for LMIC trainees to obtain graduate degrees in HIC institutions. Equal opportunities for representation of the project/study in meetings/conferences. Leadership training for principal investigators and mechanisms for safe feedback to the principal investigators on the health of their teams and partnerships with strategies in place where conflict arises.
	How can reflexivity statement around equitable partnerships be included as part of the regular grant monitoring?	Include this as a criterion in the standardised rules of conduct for representation, evaluation and monitoring of the project.
	Is there a way to include institutional responsibility for an equitable partnership as part of contract agreement?	Identify responsible named individuals within the institutions in LMIC and HIC who are funded and are tasked with monitoring the health of the partnership according to the established rules of conduct.

The stark inequities in global health have been amplified during the COVID-19 pandemic. Particularly it has shown us how much the world is connected and how much HICs risk in ignoring research needs in other countries. Current systems are not created to provide an equitable platform to all the actors in global health research. Acknowledging this is the first step in creating opportunities for coming together to create a strong, equitable and inclusive architecture for global health research [46]. This is the principle that we need to apply for transformation of the field. The risk with naming and shaming is resentment and resistance to change. Yet, the risk with skirting hard questions is that real problems will be glossed over. We are looking to generate active discussions, which begin with commitments to empathetic listening and allyship by the global North and commitments to taking greater responsibility by the global South.
